# 2,3-Dibromo-3-(5-nitro-2-fur­yl)-1-(4-nitro­phen­yl)propan-1-one

**DOI:** 10.1107/S1600536810044971

**Published:** 2010-11-13

**Authors:** Hoong-Kun Fun, Ching Kheng Quah, Shobhitha Shetty, Balakrishna Kalluraya

**Affiliations:** a X-ray Crystallography Unit, School of Physics, Universiti Sains Malaysia, 11800 USM, Penang, Malaysia; b Department of Studies in Chemistry, Mangalore University, Mangalagangotri, Mangalore 574 199, India

## Abstract

In the title compound, C_13_H_8_Br_2_N_2_O_6_, the 2-furyl ring is essentially planar, with a maximum deviation of 0.002 (2) Å. It is inclined at an angle of 33.94 (9)° to the benzene ring. Both nitro groups are slightly twisted away from their attached rings; the dihedral angles are 4.6 (2)° between the nitro group and the 2-furyl ring, and 13.72 (19)° between the nitro group and the benzene ring. In the crystal, mol­ecules are linked into chains along [110] and [1
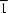
0] *via* two pairs of inter­molecular C—H⋯O hydrogen bonds, displaying *R*_2_^2^(10) ring motifs.

## Related literature

For general background to and the biological activity of nitro­furans, see: Holla *et al.* (1986 ▶, 1987 ▶, 1992 ▶). For the preparation of title compound, see: Rai *et al.* (2008 ▶). For the stability of the temperature controller used in the data collection, see: Cosier & Glazer (1986 ▶). For bond-length data, see: Allen *et al.* (1987 ▶). For hydrogen-bond motifs, see: Bernstein *et al.* (1995 ▶). For a related structure, see: Fun *et al.* (2010 ▶).
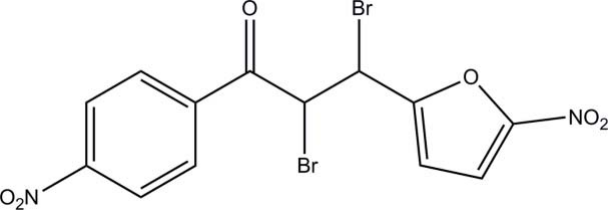


## Experimental

### 

#### Crystal data

C_13_H_8_Br_2_N_2_O_6_
*M* *_r_* = 448.03
Monoclinic, 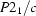

*a* = 12.1902 (2) Å
*b* = 12.2006 (2) Å
*c* = 9.9761 (2) Å
β = 96.282 (1)°
*V* = 1474.81 (5) Å^3^
*Z* = 4
Mo *K*α radiation
μ = 5.53 mm^−1^
*T* = 100 K
0.48 × 0.36 × 0.30 mm

#### Data collection

Bruker SMART APEXII CCD area-detector diffractometer
Absorption correction: multi-scan (*SADABS*; Bruker, 2009 ▶) *T*_min_ = 0.176, *T*_max_ = 0.291
22940 measured reflections
5315 independent reflections
4546 reflections with *I* > 2σ(*I*)
*R*_int_ = 0.027

#### Refinement

*R*[*F* ^2^ > 2σ(*F* ^2^)] = 0.027
*wR*(*F* ^2^) = 0.066
*S* = 1.02
5315 reflections
208 parameters
H-atom parameters constrained
Δρ_max_ = 0.84 e Å^−3^
Δρ_min_ = −0.47 e Å^−3^

### 

Data collection: *APEX2* (Bruker, 2009 ▶); cell refinement: *SAINT* (Bruker, 2009 ▶); data reduction: *SAINT*; program(s) used to solve structure: *SHELXTL* (Sheldrick, 2008 ▶); program(s) used to refine structure: *SHELXTL*; molecular graphics: *SHELXTL*; software used to prepare material for publication: *SHELXTL* and *PLATON* (Spek, 2009 ▶).

## Supplementary Material

Crystal structure: contains datablocks global, I. DOI: 10.1107/S1600536810044971/fj2362sup1.cif

Structure factors: contains datablocks I. DOI: 10.1107/S1600536810044971/fj2362Isup2.hkl

Additional supplementary materials:  crystallographic information; 3D view; checkCIF report

## Figures and Tables

**Table 1:** Hydrogen-bond geometry (Å, °)

*D*—H⋯*A*	*D*—H	H⋯*A*	*D*⋯*A*	*D*—H⋯*A*
C2—H2*A*⋯O5^i^	0.93	2.48	3.211 (2)	136
C12—H12*A*⋯O3^ii^	0.93	2.43	3.317 (2)	160

Symmetry codes: (i) 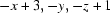; (ii) 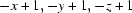.

**Fig. 1.**
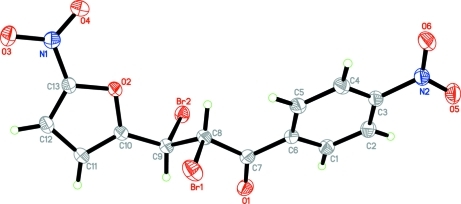
The molecular structure of the title compound showing 50% probability displacement ellipsoids for non-H atoms and the atom-numbering scheme.

**Fig. 2.**
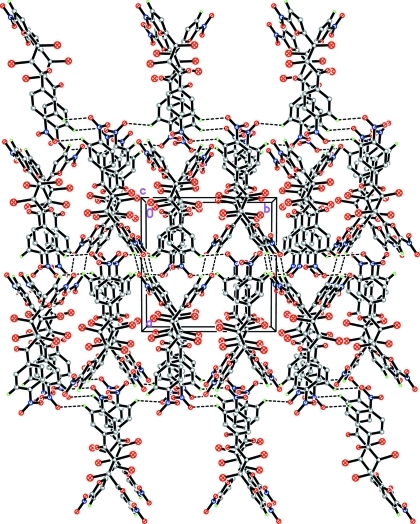
The crystal structure of the title compound, viewed along the c axis. H atoms not involved in hydrogen bonds (dashed lines) have been omitted for clarity.

## supplementary crystallographic information

### Comment

Nitrofurans are class of synthetic compounds characterized by the presence of 5-nitro-2-furyl group. The presence of nitro group in the position-5 of the molecule conferred antibacterial activity (Holla *et al.*, 1986). A number of nitrofurans have attained utility as antibacterial agents in humans and in veterinary medicine because of their broad spectrum of activity (Holla *et al.*, 1992; Holla *et al.*, 1987). 1-Aryl-3-(5-nitro-2-furyl)-2-propyn-1-ones were prepared by the hydrobromination of 2,3-dibromo-1-aryl-3-(5-nitro-2-furyl)-2-propan-1-ones in the presence of triethylamine in benzene medium. The dibromopropanones were in turn obtained by the bromination of 1-aryl-3-(5-nitro-2-furyl)-2-propen-1-ones. Acid-catalysed condensation of acetophenones with 5-nitrofuraldiacetate in acetic acid yielded the required 1-aryl-3-(5-nitro-2-furyl)-2-propen-1-ones called chalcones (Rai *et al.*, 2008).

In the title molecule (Fig. 1), the 2-furyl (O2/C10-C13) ring is essentially planar (maximum deviation = 0.002 (2) Å for atoms C11, C12 and C13) and is inclined at an angle of 33.94 (9) ° with the phenyl ring (C1-C6). Both nitro groups (N1/O3/O4 and N2/O5/O6) are slightly twisted away from the attached rings [the dihedral angles are 4.6 (2)° between nitro group and 2-fury ring and 13.72 (19)° between nitro group and phenyl ring]. Bond lengths (Allen *et al.*, 1987) and angles are within normal ranges and comparable to a related structure (Fun *et al.*, 2010).

In the crystal packing (Fig. 2), the molecules are linked into one-dimensional chains along [110] and [1-10] *via* pairs of intermolecular C2–H2A···O5 and C12–H12A···O3 hydrogen bonds, displaying R_2_^2^(10) ring motifs (Bernstein *et al.*, 1995).

### Experimental

1-(*p*-Nitrophenyl)-3-(5-nitro-2-furyl)-2-propen-1-one (0.01 mol) was dissolved in glacial acetic acid (25 ml) by gentle warming. A solution of bromine in glacial acetic acid (30% [w/v]) was added to it with constant stirring till  yellow color of the bromine persisted. The reaction mixture was kept aside at room temperature for overnight. Crystals of dibromopropanones separated out were collected by filtration and washed with ethanol and dried. It was then recrystallized from glacial acetic acid. Crystals suitable for X-ray analysis were obtained from 1:2 mixtures of DMF and ethanol by slow evaporation.

### Refinement

All H atoms were positioned geometrically and refined using a riding model with C–H = 0.93–0.98 Å and *U*_iso_(H) = 1.2 *U*_eq_(C). The highest residual electron density peak is located at 0.70 Å from Br1 and the deepest hole is located at 0.53 Å from Br2.

### Crystal data

**Table:**

C_13_H_8_Br_2_N_2_O_6_	*F*(000) = 872
*M**_r_* = 448.03	*D*_x_ = 2.018 Mg m^−^^3^
Monoclinic, *P*2_1_/*c*	Mo *K*α radiation, λ = 0.71073 Å
Hall symbol: -P 2ybc	Cell parameters from 9181 reflections
*a* = 12.1902 (2) Å	θ = 2.7–35.0°
*b* = 12.2006 (2) Å	µ = 5.53 mm^−^^1^
*c* = 9.9761 (2) Å	*T* = 100 K
β = 96.282 (1)°	Block, light yellow
*V* = 1474.81 (5) Å^3^	0.48 × 0.36 × 0.30 mm
*Z* = 4	

### Data collection

**Table:**

Bruker SMART APEXII CCD area-detector diffractometer	5315 independent reflections
Radiation source: fine-focus sealed tube	4546 reflections with *I* > 2σ(*I*)
graphite	*R*_int_ = 0.027
φ and ω scans	θ_max_ = 32.5°, θ_min_ = 2.7°
Absorption correction: multi-scan (*SADABS*; Bruker, 2009)	*h* = −18→17
*T*_min_ = 0.176, *T*_max_ = 0.291	*k* = −18→18
22940 measured reflections	*l* = −15→15

### Refinement

**Table:**

Refinement on *F*^2^	Primary atom site location: structure-invariant direct methods
Least-squares matrix: full	Secondary atom site location: difference Fourier map
*R*[*F*^2^ > 2σ(*F*^2^)] = 0.027	Hydrogen site location: inferred from neighbouring sites
*wR*(*F*^2^) = 0.066	H-atom parameters constrained
*S* = 1.02	*w* = 1/[σ^2^(*F*_o_^2^) + (0.0329*P*)^2^ + 0.6835*P*] where *P* = (*F*_o_^2^ + 2*F*_c_^2^)/3
5315 reflections	(Δ/σ)_max_ = 0.003
208 parameters	Δρ_max_ = 0.84 e Å^−^^3^
0 restraints	Δρ_min_ = −0.47 e Å^−^^3^

### Special details

**Table:**

Experimental. The crystal was placed in the cold stream of an Oxford Cyrosystems Cobra open-flow nitrogen cryostat (Cosier & Glazer, 1986) operating at 100.0 (1) K.
Geometry. All esds (except the esd in the dihedral angle between two l.s. planes) are estimated using the full covariance matrix. The cell esds are taken into account individually in the estimation of esds in distances, angles and torsion angles; correlations between esds in cell parameters are only used when they are defined by crystal symmetry. An approximate (isotropic) treatment of cell esds is used for estimating esds involving l.s. planes.
Refinement. Refinement of F^2^ against ALL reflections. The weighted R-factor wR and goodness of fit S are based on F^2^, conventional R-factors R are based on F, with F set to zero for negative F^2^. The threshold expression of F^2^ > 2sigma(F^2^) is used only for calculating R-factors(gt) etc. and is not relevant to the choice of reflections for refinement. R-factors based on F^2^ are statistically about twice as large as those based on F, and R- factors based on ALL data will be even larger.

### Fractional atomic coordinates and isotropic or equivalent isotropic displacement parameters (Å^2^)

**Table:**

	*x*	*y*	*z*	*U*_iso_*/*U*_eq_	
Br1	1.041889 (15)	0.421114 (15)	0.681600 (19)	0.03228 (5)	
Br2	0.885347 (14)	0.105613 (13)	0.491276 (17)	0.02690 (5)	
O1	1.09576 (10)	0.14526 (11)	0.72205 (13)	0.0308 (3)	
O2	0.79177 (9)	0.35369 (9)	0.44313 (11)	0.0231 (2)	
O3	0.57672 (11)	0.52110 (12)	0.31188 (15)	0.0376 (3)	
O4	0.71217 (11)	0.44726 (12)	0.21746 (13)	0.0343 (3)	
O5	1.58062 (10)	0.13853 (11)	0.42438 (14)	0.0334 (3)	
O6	1.51706 (11)	0.26900 (12)	0.29087 (14)	0.0335 (3)	
N1	0.66040 (12)	0.46327 (12)	0.31406 (15)	0.0274 (3)	
N2	1.50858 (11)	0.20428 (12)	0.38341 (14)	0.0251 (3)	
C1	1.28637 (13)	0.12333 (13)	0.59042 (16)	0.0235 (3)	
H1A	1.2691	0.0664	0.6464	0.028*	
C2	1.38425 (14)	0.12046 (13)	0.53263 (17)	0.0246 (3)	
H2A	1.4337	0.0628	0.5493	0.030*	
C3	1.40608 (12)	0.20650 (13)	0.44904 (16)	0.0219 (3)	
C4	1.33605 (14)	0.29473 (13)	0.42338 (17)	0.0253 (3)	
H4A	1.3540	0.3515	0.3676	0.030*	
C5	1.23848 (14)	0.29687 (13)	0.48241 (16)	0.0252 (3)	
H5A	1.1900	0.3555	0.4665	0.030*	
C6	1.21302 (13)	0.21085 (13)	0.56577 (15)	0.0218 (3)	
C7	1.10791 (13)	0.20769 (14)	0.62976 (16)	0.0239 (3)	
C8	1.01418 (13)	0.28532 (13)	0.57771 (16)	0.0227 (3)	
H8A	1.0158	0.2995	0.4812	0.027*	
C9	0.90304 (13)	0.23822 (13)	0.60376 (16)	0.0222 (3)	
H9A	0.9064	0.2168	0.6988	0.027*	
C10	0.80678 (13)	0.31058 (13)	0.57093 (15)	0.0222 (3)	
C11	0.72425 (13)	0.34199 (13)	0.64346 (16)	0.0245 (3)	
H11A	0.7166	0.3234	0.7324	0.029*	
C12	0.65187 (14)	0.40878 (14)	0.55717 (17)	0.0258 (3)	
H12A	0.5873	0.4425	0.5773	0.031*	
C13	0.69716 (13)	0.41271 (13)	0.43947 (16)	0.0233 (3)	

### Atomic displacement parameters (Å^2^)

**Table:**

	*U*^11^	*U*^22^	*U*^33^	*U*^12^	*U*^13^	*U*^23^
Br1	0.03186 (10)	0.03185 (9)	0.03482 (10)	−0.00803 (6)	0.01125 (7)	−0.01036 (7)
Br2	0.02422 (8)	0.02524 (8)	0.03086 (9)	0.00128 (5)	0.00126 (6)	−0.00446 (6)
O1	0.0231 (6)	0.0417 (7)	0.0277 (6)	−0.0014 (5)	0.0023 (5)	0.0128 (5)
O2	0.0209 (5)	0.0286 (5)	0.0210 (5)	0.0039 (4)	0.0068 (4)	0.0037 (4)
O3	0.0334 (7)	0.0421 (7)	0.0381 (7)	0.0164 (6)	0.0073 (6)	0.0102 (6)
O4	0.0344 (7)	0.0457 (7)	0.0241 (6)	0.0072 (6)	0.0086 (5)	0.0075 (5)
O5	0.0231 (6)	0.0371 (6)	0.0404 (7)	0.0058 (5)	0.0059 (5)	0.0039 (5)
O6	0.0285 (6)	0.0407 (7)	0.0329 (6)	0.0017 (5)	0.0104 (5)	0.0080 (5)
N1	0.0259 (7)	0.0302 (7)	0.0265 (7)	0.0032 (5)	0.0049 (5)	0.0045 (5)
N2	0.0208 (6)	0.0291 (6)	0.0255 (6)	−0.0010 (5)	0.0034 (5)	−0.0026 (5)
C1	0.0233 (7)	0.0232 (6)	0.0238 (7)	−0.0010 (5)	0.0013 (6)	0.0015 (5)
C2	0.0238 (7)	0.0234 (7)	0.0265 (7)	0.0020 (5)	0.0026 (6)	0.0003 (6)
C3	0.0193 (6)	0.0252 (7)	0.0215 (7)	−0.0010 (5)	0.0027 (5)	−0.0026 (5)
C4	0.0254 (7)	0.0276 (7)	0.0237 (7)	0.0019 (6)	0.0057 (6)	0.0033 (6)
C5	0.0254 (7)	0.0277 (7)	0.0228 (7)	0.0044 (6)	0.0045 (6)	0.0045 (6)
C6	0.0196 (7)	0.0271 (7)	0.0187 (6)	−0.0002 (5)	0.0019 (5)	0.0009 (5)
C7	0.0204 (7)	0.0302 (7)	0.0207 (7)	−0.0005 (5)	0.0009 (5)	0.0017 (5)
C8	0.0212 (7)	0.0270 (7)	0.0205 (6)	−0.0004 (5)	0.0051 (5)	−0.0008 (5)
C9	0.0202 (7)	0.0256 (7)	0.0212 (7)	0.0007 (5)	0.0037 (5)	−0.0003 (5)
C10	0.0212 (7)	0.0263 (7)	0.0198 (7)	0.0017 (5)	0.0054 (5)	0.0016 (5)
C11	0.0233 (7)	0.0294 (7)	0.0218 (7)	0.0016 (6)	0.0073 (6)	−0.0006 (6)
C12	0.0243 (7)	0.0282 (7)	0.0262 (7)	0.0049 (6)	0.0080 (6)	−0.0013 (6)
C13	0.0217 (7)	0.0251 (7)	0.0238 (7)	0.0038 (5)	0.0054 (5)	0.0015 (5)

### Geometric parameters (Å, °)

**Table:**

Br1—C8	1.9639 (16)	C3—C4	1.380 (2)
Br2—C9	1.9674 (16)	C4—C5	1.384 (2)
O1—C7	1.216 (2)	C4—H4A	0.9300
O2—C13	1.3567 (19)	C5—C6	1.395 (2)
O2—C10	1.3728 (18)	C5—H5A	0.9300
O3—N1	1.2385 (19)	C6—C7	1.493 (2)
O4—N1	1.2240 (19)	C7—C8	1.531 (2)
O5—N2	1.2260 (19)	C8—C9	1.520 (2)
O6—N2	1.2277 (19)	C8—H8A	0.9800
N1—C13	1.423 (2)	C9—C10	1.476 (2)
N2—C3	1.473 (2)	C9—H9A	0.9800
C1—C2	1.381 (2)	C10—C11	1.357 (2)
C1—C6	1.397 (2)	C11—C12	1.420 (2)
C1—H1A	0.9300	C11—H11A	0.9300
C2—C3	1.384 (2)	C12—C13	1.352 (2)
C2—H2A	0.9300	C12—H12A	0.9300
			
C13—O2—C10	104.77 (12)	O1—C7—C8	119.74 (15)
O4—N1—O3	124.87 (15)	C6—C7—C8	118.86 (13)
O4—N1—C13	118.89 (14)	C9—C8—C7	110.75 (13)
O3—N1—C13	116.23 (14)	C9—C8—Br1	109.50 (10)
O5—N2—O6	123.74 (15)	C7—C8—Br1	105.31 (10)
O5—N2—C3	118.38 (14)	C9—C8—H8A	110.4
O6—N2—C3	117.88 (14)	C7—C8—H8A	110.4
C2—C1—C6	120.76 (15)	Br1—C8—H8A	110.4
C2—C1—H1A	119.6	C10—C9—C8	115.96 (13)
C6—C1—H1A	119.6	C10—C9—Br2	109.29 (11)
C1—C2—C3	117.64 (15)	C8—C9—Br2	104.92 (10)
C1—C2—H2A	121.2	C10—C9—H9A	108.8
C3—C2—H2A	121.2	C8—C9—H9A	108.8
C4—C3—C2	123.18 (15)	Br2—C9—H9A	108.8
C4—C3—N2	118.00 (14)	C11—C10—O2	110.84 (13)
C2—C3—N2	118.82 (14)	C11—C10—C9	131.93 (15)
C3—C4—C5	118.60 (15)	O2—C10—C9	117.20 (13)
C3—C4—H4A	120.7	C10—C11—C12	106.65 (14)
C5—C4—H4A	120.7	C10—C11—H11A	126.7
C4—C5—C6	119.81 (15)	C12—C11—H11A	126.7
C4—C5—H5A	120.1	C13—C12—C11	105.13 (14)
C6—C5—H5A	120.1	C13—C12—H12A	127.4
C5—C6—C1	120.00 (15)	C11—C12—H12A	127.4
C5—C6—C7	122.11 (14)	C12—C13—O2	112.62 (14)
C1—C6—C7	117.88 (14)	C12—C13—N1	131.29 (15)
O1—C7—C6	121.40 (15)	O2—C13—N1	116.03 (14)
			
C6—C1—C2—C3	−0.6 (2)	C7—C8—C9—C10	173.89 (13)
C1—C2—C3—C4	1.2 (2)	Br1—C8—C9—C10	58.18 (16)
C1—C2—C3—N2	−178.53 (14)	C7—C8—C9—Br2	−65.45 (14)
O5—N2—C3—C4	166.33 (15)	Br1—C8—C9—Br2	178.84 (7)
O6—N2—C3—C4	−13.4 (2)	C13—O2—C10—C11	−0.01 (18)
O5—N2—C3—C2	−13.9 (2)	C13—O2—C10—C9	178.03 (14)
O6—N2—C3—C2	166.39 (15)	C8—C9—C10—C11	−128.74 (19)
C2—C3—C4—C5	−0.9 (2)	Br2—C9—C10—C11	112.98 (18)
N2—C3—C4—C5	178.81 (14)	C8—C9—C10—O2	53.73 (19)
C3—C4—C5—C6	0.0 (2)	Br2—C9—C10—O2	−64.55 (16)
C4—C5—C6—C1	0.6 (2)	O2—C10—C11—C12	0.22 (19)
C4—C5—C6—C7	−178.69 (15)	C9—C10—C11—C12	−177.43 (17)
C2—C1—C6—C5	−0.3 (2)	C10—C11—C12—C13	−0.34 (19)
C2—C1—C6—C7	179.02 (15)	C11—C12—C13—O2	0.35 (19)
C5—C6—C7—O1	−165.16 (17)	C11—C12—C13—N1	177.30 (17)
C1—C6—C7—O1	15.6 (2)	C10—O2—C13—C12	−0.22 (18)
C5—C6—C7—C8	14.6 (2)	C10—O2—C13—N1	−177.67 (14)
C1—C6—C7—C8	−164.68 (14)	O4—N1—C13—C12	−173.83 (18)
O1—C7—C8—C9	−26.5 (2)	O3—N1—C13—C12	6.2 (3)
C6—C7—C8—C9	153.73 (14)	O4—N1—C13—O2	3.0 (2)
O1—C7—C8—Br1	91.78 (16)	O3—N1—C13—O2	−176.98 (15)
C6—C7—C8—Br1	−87.98 (14)		

### Hydrogen-bond geometry (Å, °)

**Table:**

*D*—H···*A*	*D*—H	H···*A*	*D*···*A*	*D*—H···*A*
C2—H2A···O5^i^	0.93	2.48	3.211 (2)	136
C12—H12A···O3^ii^	0.93	2.43	3.317 (2)	160

Symmetry codes: (i) −*x*+3, −*y*, −*z*+1; (ii) −*x*+1, −*y*+1, −*z*+1.
